# Crystallinity-Dependent Thermoelectric Properties of a Two-Dimensional Coordination Polymer: Ni_3_(2,3,6,7,10,11-hexaiminotriphenylene)_2_

**DOI:** 10.3390/polym10090962

**Published:** 2018-08-31

**Authors:** Yoshiyuki Nonoguchi, Dai Sato, Tsuyoshi Kawai

**Affiliations:** 1 Division of Materials Science, Nara Institute of Science and Technology, Ikoma 630-0192, Japan; 2 JST PRESTO, Kawaguchi 332-0012, Japan; 3 Graduate School of Materials Science, Nara Institute of Science and Technology, Ikoma 630-0192, Japan; dai.sato@trichemical.com

**Keywords:** coordination polymers, thermoelectric properties, molecular conductors

## Abstract

The evaluation of thermoelectric properties has recently become a standard method for revealing the electronic properties of conducting polymers. Herein we report on the thermoelectric properties of a two-dimensional coordination polymer pellets. The pellets of Ni_3_(2,3,6,7,10,11-hexaiminotriphenylene)_2_, which has recently been developed, show n-type thermoelectric transport, dependent on crystallinity. The present results provide systematic feedback to the guideline for high-performance molecular thermoelectric materials.

## 1. Introduction

Due to the rapid progress of synthesis technology and increased interests in unique transport and thermoelectric properties of atomically thin two-dimensional materials, these have recently been explored. Such two-dimensional films typically consist of sp^2^ carbon (graphenes), black phosphorous, and transition metal dichalcogenides (TMDC) as well as typical oxide semiconductors [1]. Thermoelectric properties reflect the electronic structure around the Fermi level, which has been used for the investigation of transport phenomena. In this context, low-dimensional materials having unique electronic structures are preferred objects for thermoelectric studies.

Low-dimensional materials can be designed by organic and inorganic synthetic chemistry. Representative examples include charge transfer complexes whose electronic properties vary from molecular metals to semiconductors [2,3,4]. In these previous works, thermoelectric properties were evaluated mostly for determining carrier polarity and estimating band structures around the Fermi level. Recently, several groups have reported that one-dimensional metal coordination polymers (CPs) exhibit excellent thermoelectric properties. The 1,1,2,2-ethenetetrathiolate (ett)-containing polymers, poly[Na_x_(Ni-ett)] and poly[Cu_x_(Cu-ett)], showed n-type and p-type thermoelectric properties with the power factor of ca. 30, and 15 μW·m^−1^·K^−2^, respectively [5]. This report was based on the discovery of high conductivity CPs.

**Figure 1:**
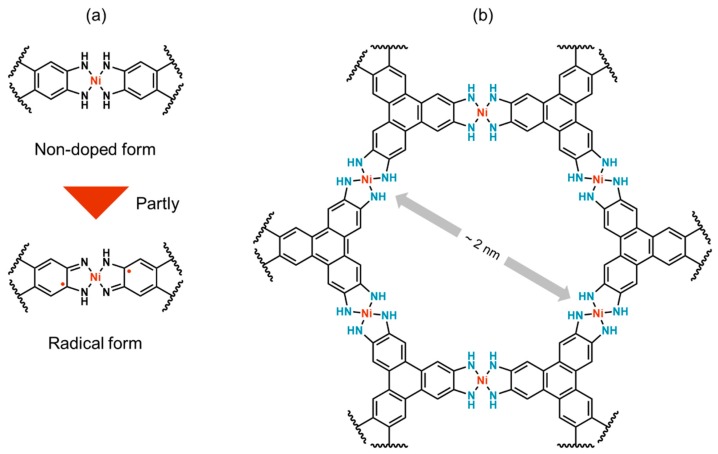
(**a**) The formation of the radical state that generates charged carriers. (**b**) The representative fragment of Ni_3_(HITP)_2_.

One-dimensional CPs based on benzene tetraamine (BTA) are reported to exhibit moderate electrical conductivity, which has recently been used for application in the fabrication of a resistive random access memory [6]. Recently, conducting two-dimensional (2D-) CPs have been synthesized, which stimulates their electron transfer and transport studies. The components of framework determine their electronic and structural properties such as bandgap, and porosity. Among them, several 2D-CPs seem to show metallic conduction. Particularly, the small crystalline sheet of Ni_3_(HITP)_2_ (HITP = 2,3,6,7,10,11-hexaiminotriphenylene) shows the relatively high conductivity of 40 S·cm^−1^ [7] and has been proposed to act as an electrocatalyst [8]. The high conductivity in these studies relies on the formation of a stable radical form (Figure 1a). So far, the transport properties of Ni_3_(HITP)_2_ have been yet unexplored well. In this work, we examine the thermoelectric properties of Ni_3_(HITP)_2_ pellets prepared using several Ni salts. We reveal that all these pellets show the negative and small Seebeck coefficient, that is, n-type metallic characteristics. Additionally, the conductivity is dependent on the crystallinity determined using X-ray diffraction (XRD) patterns. These results suggest the Ni_3_(HITP)_2_ is intrinsically a molecular metal, and support the validity to use it as various electron-transporting components including catalyst.

## 2. Materials and Methods

**Scheme 1:**
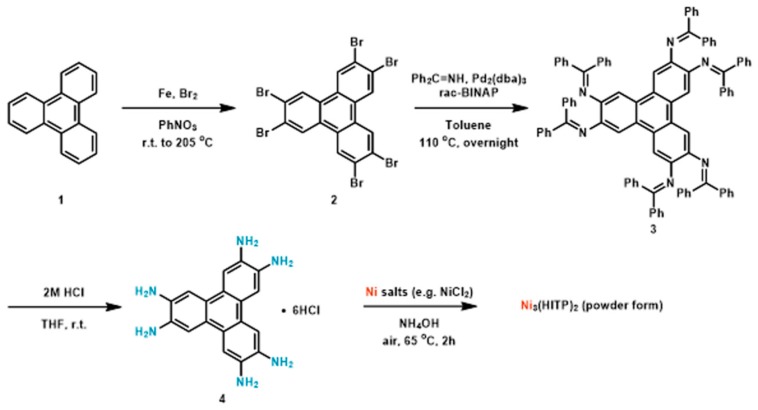
A synthetic route to Ni_3_(HITP)_2_.

We synthesized Ni_3_(HITP)_2_ by following reported procedures (Scheme 1) [7], through the preparation of each intermediates (2–4) [9,10]. After the bromination of triphenylene [9], we synthesized the intermediate 3 (84%, MALDI-TOF MS *m*/*z* 1303.4; ^1^H NMR (300 MHz, DMSO) δ7.11–7.13 (m, 12H), 7.20 (s, 6H, triphenylene CH), 7.27–7.29 (m, 20H), 7.40–7.48 (m, 20H), 7.60–7.63 (m, 13H)) [10]. The hydrolysis of 3 resulted in the preparation of 2,3,6,7,10,11-hexaiminotriphenylene hydrochloric acid salts (80%, DART MS *m*/*z* 319 [M + H]^+^; ^1^H NMR (300 MHz, DMSO) δ3.43 (brs, 18H), 7.85 (s, 6H)). Finally, we obtained Ni_3_(HITP)_2_ as black powders (60%). The structural characterizations of each intermediate and Ni_3_(HITP)_2_ were performed by NMR (JEOL JNM-AL300, Tokyo, Japan), mass spectroscopy (DART, JEOL JMS-Q1000TD; MALDI-TOF MS, Bruker Japan Autoflex II, Yokohama, Japan), transmission electron microscopy (JEOL JEM-3100FEF, Tokyo, Japan), X-ray diffraction (Rigaku RINT-TTR III/NM, Akishima, Japan), and X-ray photoelectron spectroscopy (XPS, ULVAC-PHI, Inc. PHI 5000 VersaProbe II, Chigasaki, Japan). DC electrical conductivity was measured using the 4-point probe method (Mitsubishi Chemical Loresta GP Model MCP-T610, Yamato, Japan). Thermoelectric voltage was recorded using a Seebeck coefficient measurement system (MMR technologies K20SB100-3R, San Jose, CA, USA) with a high-impedance amplifier [11,12,13,14,15]. Test specimens were prepared by soft-pressing at 18.5 MPa (~3 ton).

## 3. Results

### 3.1. Structural Characterization

**Figure 2:**
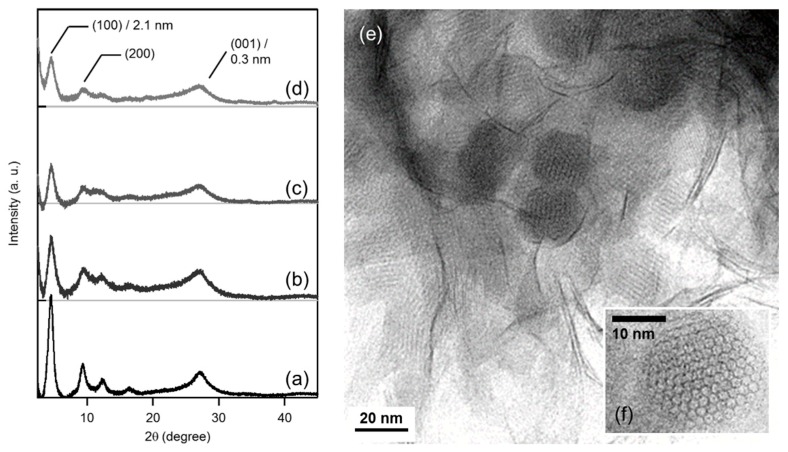
XRD patterns of Ni_3_(HITP)_2_ powders prepared using (**a**) NiCl_2_, (**b**) Ni(NO_3_)_2_, (**c**) NiBr_2_, and (**d**) Ni(OTf)_2_. (**e**) Representative TEM images of Ni_3_(HITP)_2_. An inset shows a magnified image.

The XRD patterns of prepared powders indicated the highly ordered hexagonal lattice with intense (100), (200), and (001) peaks (Figure 2a–d). In detail, these structures were assigned to the eclipsed or slipped-parallel structures, or both, corresponding to the first report on their preparation [7]. We examined the effects of Ni salts species (NiCl_2_, Ni(NO_3_)_2_, NiBr_2_, and Ni(OTf)_2_) on the crystallinity of final products. In the basic aqueous condition, NiCl_2_ was likely to afford sharp XRD patterns, indicating the most crystalline solids as discussed later. Such difference might result from the solubility and reactivity of Ni^2+^ feedstocks. TEM observation revealed the highly ordered mesoporous structures with hexagonal lattices (Figure 2e). The magnified image more clearly displays the hexagonally ordered mesopores (Figure 2f). Additionally, tens-nm-long mesochannel structures formed, indicating moderate c-axis stacking. The averaged pore diameter found in TEM images was approximately 2.0 nm, and this size was consistent with the molecular modeling [7] (Figure 1b) and the (100) peak (*d-spacing* = 2.1 nm) observed in the XRD profile (Figure 2a). In addition to such mesochannel bundles, the powders were also likely to consist of laterally grown nanosheets as the sheet edges were observed. This lateral growth is consistent with the strong (100) peak in the powder XRD. Further elemental analyses were performed using XPS (Figure S1).

We prepared pellets by pressing the powder and evaluated the electrical conductivity of approximately 1 S·cm^−1^, which mostly reproduces the previous study [7]. Conduction pathway is attributed mostly to laterally grown framework, and in this work, such lateral electron transport in single crystals was limited within several tens nanometer. Therefore, an approach to promoting lateral crystal growth would be required for further improving electrical conductivity in the future work.

### 3.2. Transport Study

Soft-pressed pellets showed relatively low electrical resistance, which allowed to evaluate the Seebeck coefficient. The Seebeck coefficient is a generated voltage across the temperature gradient. For common metals, the relationship of the Seebeck coefficient (*α*) with electrical conductivity (*σ*) can be expressed as,

(1) $$\alpha =\frac{\pi^{2}}{3}\;\frac{k_{B}^{2}T}{q}\;\frac{1}{\sigma }\;\frac{\delta \sigma }{\delta \varepsilon }\;{|}_{\varepsilon =E_{F}}$$

where *k_B_* is the Boltzmann constant, *q* is the elemental charge, *T* is the absolute temperature, and *E_F_* is the Fermi energy. This equation is known as the Mott formula, and simply indicates the trade-off relationship between *σ* and *α* [16].

**Figure 3:**
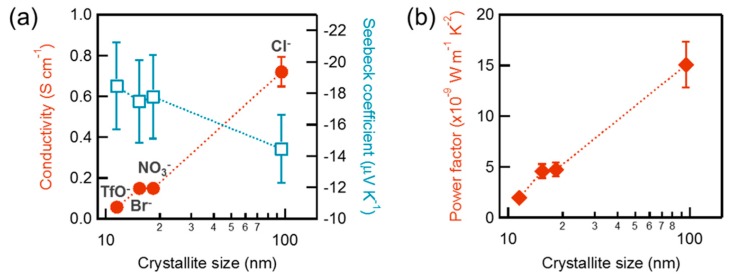
(**a**) Electrical conductivity, the Seebeck coefficient, and (**b**) power factor of Ni_3_(HITP)_2_ pellets as a function of crystallite size.

First, the Seebeck coefficients observed here were small (|*α*| < 20 μV·K^−1^), suggesting the dominant contribution of metallic conduction in Ni_3_(HITP)_2_. These values were indeed close to the typical Seebeck coefficient of metals [e.g., −10 μV·K^−1^ for palladium, and +14 μV·K^−1^ for iron]. Second, the sign of the Seebeck coefficient was negative, indicating n-type transport. Furthermore, we found the tunable thermoelectric properties of Ni_3_(HITP)_2_ pellets dependent on salt species used in the synthesis. We used four nickel salts (NiCl_2_, Ni(NO_3_)_2_, NiBr_2_, and Ni(OTf)_2_) as feedstocks, where NiCl_2_-derived Ni_3_(HITP)_2_ pellets demonstrated the highest electrical conductivity (~0.7 S·cm^−1^, Figure 3a). As the electrical conductivity increased, the Seebeck coefficient decreased gradually. We further considered the effect of the crystalline size (*D*), which is conventionally evaluated by the Scherrer’s equation as follows,

(2) $$D=\frac{K\lambda }{Bcos\theta }$$

where *B* is the observed line width, *λ* is the wavelength of incident X-ray, and *K* is the Scherrer constant. We found that the large *D* value indicating high crystallinity leads to an increasing in conductivity and a decreasing in the negative Seebeck coefficient as shown in Figure 3a. The analyses of a (100) peak with the Scherrer’s equation revealed that the *D* value can be modulated from 11 nm to 95 nm, using the different Ni salt feedstocks (Figure 2a–d). The increasing *D* value was then significantly influential to electrical conductivity, rather than the Seebeck coefficient. As a result, the Ni_3_(HITP)_2_ pellet derived from NiCl_2_ exhibited the highest power factor (*σα*^2^) of 1.5 × 10^−8^ W·m^−1^·K^−2^ (Figure 3b) with the largest *D* value. We could not perform further reliable chemical doping to the powdery samples since the pellets were fragile.

## 4. Discussion

Some previous systematic studies on micro-scale conductivity has revealed that the 2D-CPs including dithiolene-based CPs [17,18,19,20] showed the metal-like temperature dependence of electrical conductivity exceeding 100 S·cm^−1^. Particularly, copper complexes showed the small temperature dependence of electrical conductivity and very small Seebeck coefficients ranging from −4 to −10 μV·K^−1^, which suggested metallic conduction associated with the variable-range hopping mode [20]. On the other hand, Ni_3_(HITP)_2_ nanosheets showed the positive temperature dependence of electrical conductivity, and this has been attributed to semiconductor-like transport [7]. Foster has predicted that the pristine Ni_3_(HITP)_2_ is intrinsically a metal, but due to defect- and slipping-related grain boundaries, most bulk solids could show semiconductor-like transport properties [21]. Such the opened band-gap energy was estimated to be as large as 100 meV. Thermoelectric properties reflect the band structure at the Fermi level especially energy gradient of the density of state at the Fermi-level. Since deep doping is expected in the present polycrystalline pellets, the observed thermoelectric properties are likely to highlight the metallic characteristics of Ni_3_(HITP)_2_, which would veil the practical semiconductor-like characteristics [7]. In this consideration the larger grain size seems to support higher conductivity with less charge carrier scattering. High crystallinity could suppress the Seebeck coefficient as a result of the broadened density-of-state (DOS) distribution. Additionally, Ni_3_(HITP)_2_ showed n-type transport, which is consistent with the oxidative formation of representative Ni^2+^/Ni^3+^ mixed valence compounds from Ni^2+^ [22], and the formation of HITP anion radicals.

## 5. Conclusions

We have examined the thermoelectric properties of Ni_3_(HITP)_2_ pellets dependent on crystallinity. The Seebeck coefficient ranged from −10 to −20 μV·K^−1^, which seemed to reflect the metallic characteristics of Ni_3_(HITP)_2_. Crystallinity was crucial for exploiting high electrical conductivity, contributing to an increase in the thermoelectric power factor. The present work gives an insight into the design of molecular thermoelectric materials.

## Supplementary Materials

The following are available online at http://www.mdpi.com/2073-4360/10/9/962/s1, Figure S1: XPS of a Ni_3_(HITP)_2_ powder.
