# Supplementary material for: Crystallinity-Dependent Thermoelectric Properties of a Two-Dimensional Coordination Polymer: Ni3(2,3,6,7,10,11-hexaiminotriphenylene)2
Source: Polymers (Basel). 2018 Aug 31;10(9):962. doi: 10.3390/polym10090962 (PMC6404083; doi:10.3390/polym10090962)
Supplement: Supplementary file 1 [file polymers-10-00962-s001.pdf]

Crystallinity-dependent Thermoelectric Properties of a Two-dimensional Coordination Polymer: Ni_3(2,3,6,7,10,11-hexaiminotriphenylene)$_2$

Yoshiyuki Nonoguchi [1,2,*], Dai Sato [3] and Tsuyoshi Kawai [1*]

1. Division of Materials Science, Nara Institute of Science and Technology, Ikoma 630-0192, Japan
2. JST PRESTO, Kawaguchi 332-0012, Japan
3. Graduate School of Materials Science, Nara Institute of Science and Technology, Ikoma 630-0192, Japan

*****Correspondence: nonoguchi@ms.naist.jp, tkawai@ms.naist.jp; Tel.: +81-743-72-6181

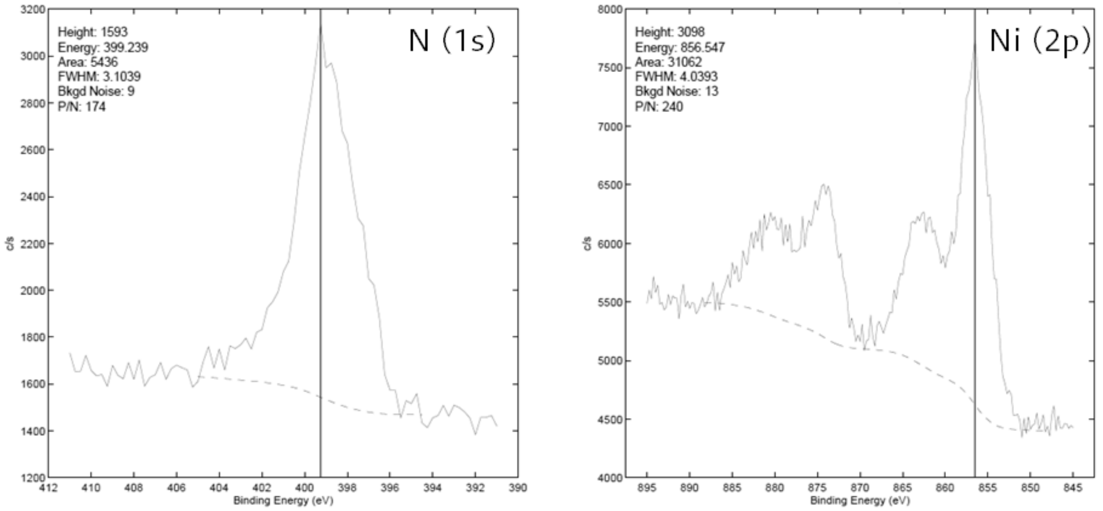

Figure S1. XPS of a $Ni_3(HITP)_2$ powder.

We used XPS to check the composition. We have observed the single type of Ni and N, and no trance of Cl. This fact is corresponding to the previous report [ref.7].
